# Adherence to the MIND diet is associated with 12-year all-cause mortality in older adults

**DOI:** 10.1017/S1368980020002979

**Published:** 2022-02

**Authors:** Janie Corley

**Affiliations:** Lothian Birth Cohorts Group, Department of Psychology, University of Edinburgh, 7 George Square, Edinburgh, EH8 9JZ, UK

**Keywords:** MIND diet, Mediterranean diet, Dietary patterns, Mortality, Older adults

## Abstract

**Objective:**

To prospectively evaluate the association of three dietary patterns: the MIND (Mediterranean-DASH diet intervention for Neurodegenerative Delay) diet; a Mediterranean-type diet and a traditional diet, with all-cause mortality over a 12-year period in an older sample.

**Design:**

A longitudinal birth cohort study. We ascertained dietary patterns using FFQ data at baseline (2004–2007) and mortality using linkage data. Cox regression was used to estimate mortality hazard ratios (HR) with adjustment for confounders.

**Setting:**

The Lothian Birth Cohort 1936 (LBC1936) study in Edinburgh, Scotland.

**Participants:**

Dietary patterns were ascertained in 882 participants, mean age 69·5 (±0·8) years, at baseline. During the 12-year follow-up (to October 2019), 206 deaths occurred.

**Results:**

In the basic-adjusted model, all three dietary patterns were significantly associated with mortality, the MIND diet and Mediterranean-type diet with a lower risk and the traditional diet with a higher risk. In fully adjusted models, MIND diet score was inversely related to all-cause mortality (HR 0·88; 95 % CI 0·79, 0·97) such that the risk of death was reduced by 12 % per unit increase in MIND diet score. Participants in the top compared with the bottom third of MIND diet score had a 37 % lower risk of death (HR 0·63; 95 % CI 0·41, 0·96). No significant associations with the Mediterranean-type or traditional dietary patterns were observed in the final multivariate model.

**Conclusions:**

Our findings suggest that closer adherence to the MIND diet is associated with a significantly lower risk of all-cause mortality, over 12 years of follow-up, and may constitute a valid public health recommendation for prolonged survival.

Quality of diet is fundamental to health. Epidemiological evidence suggests that the Mediterranean diet, characterised by an abundance of fruits, vegetables, fish, legumes, olive oil and wholegrains, is an optimal diet for promoting longevity and preventing chronic ageing-related disease^(1)^. Conversely, eating a more traditional or processed diet has been linked to poorer health outcomes and an increased risk of mortality^(2–4)^. Assessing the relationship between diet and risk of mortality is of scientific interest because it can shed light on the possible health benefits gained by adhering to certain dietary patterns and therefore has the potential to translate into public health recommendations for reducing the risk of premature death.

Systematic reviews and meta-analyses of prospective studies report clear and consistent evidence that individuals who adhere to a Mediterranean diet are less likely to die^(5,6)^. Averaged across fourteen prospective cohort studies, a two-unit increase in Mediterranean diet score was associated with decreased mortality from any cause by approximately 8 % (pooled hazard ratio (HR) 0·92; 95 % CI 0·91, 0·93)^(6)^. Recently, a larger meta-analysis of twenty-nine prospective cohort studies reported similar findings, supporting an inverse association (pooled HR 0·90, 95 % CI 0·89, 0·91), whereby a two-unit increase in the adherence to a Mediterranean diet was associated with a 10 % lower risk of all-cause mortality^(7)^. However, the magnitude of the ‘protective’ effect varies markedly between individual studies. For example, the SUN cohort based in Spain reported a substantial 17 % reduced risk of mortality per two-unit increase in Mediterranean score and a 62 % reduced risk in the high compared with the low adherence groups^(8)^. Evidence suggests stronger associations in Mediterranean populations where the consumption of the beneficial components of the Mediterranean diet is higher^(7,9)^.

Few studies have focused on older adults. In the only meta-analysis of the Mediterranean diet with survival in the elderly (≥65 years, *n* 11 738) to date, a two-unit increase in Mediterranean diet score was associated with a 10 % lower risk of all-cause mortality^(10)^. This pooled survival estimate, averaged across seven prospective studies, is comparable with those reported among the mixed age cohorts, as above^(5–7)^. However, in one UK study, a healthy dietary pattern was protective against mortality in the elderly only when participants practised at least one other healthy lifestyle factor such as never smoking, occasional drinking or physical activity^(11)^.

The MIND (Mediterranean-DASH Intervention for Neurodegenerative Delay) diet^(12)^ has been developed in recent years and is based on elements of the Mediterranean diet and the DASH (Dietary Approaches to Stop Hypertension) diet. The MIND diet shares many food groups with both diets and emphasises plant-based foods but with modifications reflecting the best scientific evidence for nutrition and the ageing brain. As such, it specifically promotes the consumption of green leafy vegetables and berries, but not overall fruit intake, for which the overall evidence is weak^(13)^, and limited intakes of animal-based and high-saturated fat foods. Several studies have reported that the MIND diet is associated with slower cognitive decline^(12)^, reduced rates of cognitive impairment^(14)^, Alzheimer’s disease^(15)^ and Parkinson’s disease^(16)^. In some studies, the MIND diet score was a better predictor of cognitive decline^(12)^ and incidence of cognitive impairment^(14)^ compared with the Mediterranean diet.

The MIND diet may be a relevant target for mortality research because it was originally designed to provide dietary guidelines with the specific intention of addressing neuroprotection and is a combination of two diets that have been shown to lower the risk of hypertension, heart attack and stroke^(17,18)^. Yet, to the best of our knowledge, there has been no attempt to evaluate the MIND diet in relation to mortality and hence validate its presumed efficacy as a public health intervention strategy.

Thus, in the first known study to investigate the MIND diet with mortality, our aim was to evaluate associations between adherence to the MIND diet (using an *a priori* score developed by Morris *et al*.)^(12)^ and mortality risk over 12 years in a narrow-age cohort of older Scottish men and women. We additionally examine mortality associations with two further dietary patterns – a Mediterranean-type dietary pattern and a traditional pattern – both were previously derived for this sample at baseline using a data-driven (*a posteriori*) approach.

## Methods

### Study population

The Lothian Birth Cohort 1936 (LBC1936) is a group of individuals born in 1936 who took part in the 1947 Scottish Mental Survey, a nationwide survey of intelligence, when they were aged 11 years^(19)^. Individuals who took the original test, and living in the Lothians region of Scotland, were contacted almost 60 years later, of whom 1091 were recruited to the LBC1936. Since baseline (2004–2007, mean age = 69·5 years), participants have subsequently attended additional follow-up assessments at ages 73 (wave 2, *n* 866), age 76 (wave 3, *n* 697), age 79 (wave 4, *n* 550), age 82 (wave 5, *n* 431) and the study is ongoing. Extensive phenotypic data have been collected including cognitive, neuroimaging, health, medical, genetic and epigenetic, psychosocial and lifestyle measures. Full recruitment and testing procedures of the LBC1936 are described elsewhere^(20)^.

### Exclusion criteria

At baseline, all participants (*n* 1091) were invited to complete a FFQ at home for return by post. A total of 124 FFQ (11 %) were not completed (ninety-eight were not returned and twenty-six were returned blank). Of the 967 returned FFQ, thirty-nine were excluded with >10 missing items, according to standard FFQ protocol, and a further forty-six were excluded due to having extreme energy intakes, defined as <2·5th or >97·5th centile. Thus, 882 participants provided usable dietary information at baseline.

### Dietary assessment

Dietary intake was assessed at baseline using a semi-quantitative 168-item Scottish Collaborative Group FFQ^(21)^. Participants were asked to indicate how often they consumed a given item (using a common unit or portion size) according to a nine-category frequency scale ranging from ‘rarely or never’ to ‘7 or more times/d’. The FFQ has been validated against weighed food intake in British populations^(22)^.

#### The MIND diet

The MIND score^(12)^ comprises fifteen dietary components: ten brain healthy foods (green leafy vegetables, other vegetables, nuts, berries, beans, wholegrains, fish, poultry, olive oil and wine) and five less healthy foods (red meats, butter/margarine, cheese, pastries and sweets and fried/fast food). Olive oil consumption was scored 1 if used as the primary oil. For all other diet score components, the frequency of consumption of each food item associated with that component was summed and then assigned a concordance score of 0, 0·5 or 1. The total MIND diet score was calculated by adding the individual component scores. The Scottish Collaborative Group FFQ did not include questions for butter/margarine consumption as separate items, so the maximum score possible in the current sample was 14, with higher scores indicating greater adherence to the MIND diet.

#### The Mediterranean-type diet and traditional diet

Dietary patterns in the LBC1936 at baseline were derived previously, by applying principal component analysis to all FFQ items. Full details can be found in Mõttus *et al*.^(23)^. Principal component analysis is a widely used exploratory method in epidemiology to derive population-specific dietary patterns from habitual diet^(24,25)^. Briefly, components were rotated by a Varimax procedure resulting in non-correlated factors to facilitate interpretability. Components were retained based on an eigenvalue >1·25, Scree plots and interpretability. A cut-off of 0·30 was used to determine factor loadings included in each pattern. Two major patterns were extracted and labelled according to the types of foods with the highest factor loadings. The first component was characterised by food loadings (indicating greater consumption) of vegetables, fish, poultry, legumes, pasta, rice, water, tomato-based sauces, olive oil and salad dressing. This component captured a healthy diet and included loadings from foods typical of a Mediterranean diet and, thus, labelled ‘a Mediterranean-type’ dietary pattern. A second component was characterised by food loadings from meat and processed meats such as chicken pies, pasties and sausage rolls, tinned vegetables, peas or beans, carrots, baked beans, bottled sauces, mashed potatoes, custard or other sweet sauces, milk-based puddings and drinking less ground (filter, espresso or cappuccino) coffee. Overall, this diet typifies a traditional Scottish diet which is higher in processed and convenience foods, and thus, this component was labelled a traditional dietary pattern. Factor scores for both dietary patterns were calculated for each participant by summing the intakes of the food groups weighted by their factor loadings. The higher the score, the closer the diet to the dietary pattern, and the lower the score, the further the diet from the dietary pattern.

### Assessment of potential confounders

Covariates were assessed at the baseline visit when participants underwent interviews with trained psychologists and nurses. Total energy intake (kcal/d) was calculated using full FFQ data. Age 11 IQ was derived from scores obtained from 1947 Scottish Mental Survey records and converted to a standard IQ-type score (where mean = 100, sd = 15). Education was assessed using years of formal full-time education. Own socio-economic status (SES) was coded from 1 (professional) to 5 (unskilled) and calculated from highest obtained occupation data, using the Registrar General’s Classification of Occupations 1980^(26)^. Father’s SES was coded from 1 (professional) to 5 (unskilled) according to the 1950 Classification of Occupations^(27)^. Smoking status was categorised into never smoker, former smoker and current smoker. Depressive symptoms were measured using the Hospital Anxiety and Depression Scale depression subtest^(28)^, which has a score range from 0 to 21. Physical activity (level) was derived from responses to a questionnaire item which asked: ‘What level of physical activity do you mainly do?’ and responses were on a six-point scale: (1) moving only in connection with necessary (household) chores; (2) walking or other outdoor activities 1–2 times/week; (3) walking or other outdoor activities several times a week; (4) exercising 1–2 times/week to the point of perspiring and heavy breathing; (5) exercising several times/week to the point of perspiring and heavy breathing and (6) keep fit/heavy exercise or competitive sport several times/week. BMI was calculated as weight (in kg)/height (in m)^2^; height and weight measures were taken by nurses at the time of assessment. History of disease (hypertension, CVD, diabetes and stroke) was binary coded as no (0) and yes (1).

### Mortality ascertainment

Mortality status was obtained via linkage data from the National Health Service Central Register, provided by the General Register Office for Scotland (now National Records of Scotland). Participants were followed from baseline assessment date (between 2004 and 2007) to the end of the follow-up period (October 2019). During this period, the LBC1936 study was informed of 277 deaths (25 % of cohort), 206 of whom had provided dietary data at baseline.

### Statistical analyses

Participants were divided into tertiles of diet scores for descriptive purposes only. Participant characteristics were compared across tertiles using ANOVA or *χ* ^2^ test for categorical variables.

Cox proportional hazard regression models were used to estimate the effects of diet score (analysed as continuous variables) on all-cause mortality. Relative risks were estimated as HR with 95 % CI. In the Cox models, basic-adjusted (age, sex and energy intake) and multivariate-adjusted HR were calculated for each of the three diet scores. In a second (+ demographic/lifestyle-adjusted) model, we additionally included age 11 IQ, education, own SES, father’s SES, smoking status, depression and physical activity. In a third (+ health-adjusted) fully adjusted model, we additionally included BMI, hypertension, CVD, diabetes and stroke.

*Post hoc* analyses repeated the same models but using a categorical measure of diet (according to tertiles of adherence) if the continuous variable was previously significant in the Cox regression models. We used the lowest tertile of adherence (i.e. least adherent) as the reference group.

Survival to follow-up date (October 2019) was assessed based on the day of initial assessment at baseline. All statistical analyses were done in SPSS (version 25; SPSS Inc.), and a *P* < 0·05 was considered significant.

## Results

Baseline dietary data were available for 882 participants (51·8 % female). The mean age of participants at baseline was 69·5 (±0·8) years. During a mean follow-up time of 12·1 (±3·2) years (median = 13·2 years), there were 206 deaths (41·7 % female). Baseline characteristics of participants according to survival status are presented in Table 1. Participants who died over the follow-up period were more likely to be male, a current or former smoker, less physically active, and to have a lower age 11 IQ score, a lower own SES and father SES, more depressive symptoms, a higher BMI and a self-reported history of disease (hypertension, CVD, diabetes and stroke).

**Table 1:** Characteristics of participants (*n* 882) according to mortality status, Lothian Birth Cohort 1936 study

Covariates	Alive	Deceased	*P* value
*n*	676	206	
Age (years)	69·6	69·5	0·08
Female sex (%)	54·9	41·7	0·001
Energy intake (kcal/d)*	1932	1878	0·31
Age 11 IQ	100·7	97·9	0·03
Education (years)	10·8	10·8	0·66
Depressive symptoms†	2·64	3·27	0·004
BMI	27·5	28·5	0·003
Own SES (%)			0·001
Classes 1 + 2 (prof)	59·4	49·5	
Class 3 (non-manual)	24·4	21·8	
Class 3·5 (manual)	13·0	24·8	
Classes 4 + 5 (manual)	3·2	4·0	
Father SES (%)			0·001
Groups 1 + 2 (prof)	28·0	26·6	
Group 3 (non-manual)	56·4	55·2	
Groups 4 + 5 (manual)	15·6	18·2	
Smoking (%)			<0·001
Never smoker	51·7	31·6	
Former smoker	42·0	45·1	
Current smoker	6·2	23·3	
Physical activity‡ (%)			0·003
Levels 1 + 2 (low)	23·7	35·5	
Levels 3 + 4 (med)	65·6	55·7	
Levels 5 + 6 (high)	10·7	8·9	
Hypertension (% yes)	37·0	47·1	0·009
CVD (% yes)	21·4	33·0	0·001
Diabetes (% yes)	5·2	14·1	<0·001
Stroke (% yes)	3·0	8·3	0·001

SES, socioeconomic status; prof, professional.

* To convert kcal to kJ, multiply it by 4·184.

† Depressive symptoms calculated using the Hospital Anxiety and Depression Scale – Depression subscale score, (range 1–21).

‡ Physical activity levels: 1, moving only in connection with necessary (household) chores; 2, walking or other outdoor activities 1–2 times/week; 3, walking or other outdoor activities several times a week; 4, exercising 1–2 times/week to the point of perspiring and heavy breathing; 5, exercising several times/week to the point of perspiring and heavy breathing; 6, keep fit/heavy exercise or competitive sport several times/week.

Table 2 presents the baseline characteristics of participants according to tertiles of each dietary predictor (MIND, Mediterranean-type and traditional diet pattern) score. Higher MIND (*P* < 0·001) and Mediterranean-type (*P* = 0·005) diet scores, and lower traditional (*P* = 0·008) diet scores, were associated with fewer deaths to follow-up. Higher MIND diet adherence (range 0–13, out of a maximum possible score of 14) was associated with being female, and having a higher age 11 IQ, more education, a more professional SES, less smoking and less depressive symptoms. Higher Mediterranean-type diet adherence was associated with being younger, a higher overall energy intake, higher age 11 IQ and more education, a more professional own SES and father SES, less smoking, less depressive symptoms and more exercise. In contrast, higher traditional diet adherence was associated with being older, male, a higher overall energy intake, a lower age 11 IQ, less education, a lower own SES and father SES, more smoking, more depressive symptoms, less exercise and a history of stroke. We note, here, that unlike the Mediterranean-type diet and traditional diet pattern, there was no association between MIND diet adherence and energy intake.

**Table 2:** Associations between baseline (2004–2007) characteristics and tertiles of the MIND, Mediterranean-type and traditional diet scores, Lothian Birth Cohort 1936 study

	MIND diet			Mediterranean-type diet			Traditional diet		
Covariates	Low	High	*P*_trend_	Low	High	*P*_trend_	Low	High	*P*_trend_
Age (years)	69·6	69·4	0·06	69·6	69·4	0·004	69·4	69·6	0·003
Female sex (%)	39·0	66·7	<0·001	47·6	55·8	0·14	61·9	42·8	<0·001
Energy (kcal/d)*	1901	1886	0·93	1691	2113	<0·001	1703	2139	<0·001
Age 11 IQ	99·3	102·5	0·008	98·7	103·7	<0·001	106·3	97·9	<0·001
Education (years)	10·6	11·0	<0·001	10·5	11·1	<0·001	11·2	10·5	<0·001
Own SES† (% prof)	45·7	66·5	<0·001	39·9	71·6	<0·001	73·6	43·7	<0·001
Father SES‡ (% prof)	22·3	31·2	0·26	17·2	33·6	0·001	36·8	23·2	0·001
Current smoker (%)	18·1	4·3	<0·001	14·3	4·8	0·003	7·1	15·0	0·001
Depressive symptoms§	3·0	2·4	0·001	2·9	2·4	0·04	2·4	3·0	0·005
Physical activity‖ (% low)	31·1	21·9	0·01	33·0	20·5	<0·001	22·5	26·5	0·02
BMI	27·8	27·2	0·20	27·7	27·4	0·59	26·9	28·2	<0·001
Hypertension (% yes)	39·4	39·6	0·98	42·5	35·7	0·24	36·1	45·0	0·08
CVD (% yes)	25·9	23·9	0·64	25·2	23·8	0·88	23·1	26·9	0·40
Diabetes (% yes)	6·7	7·1	0·84	9·2	4·8	0·11	5·4	8·8	0·28
Stroke (% yes)	5·3	3·9	0·41	5·1	3·1	0·45	1·7	5·8	0·03
Deaths (% yes)	32·3	16·1	<0·001	29·9	20·0	0·005	17·7	28·6	0·008

SES, socio-economic status; prof, professional.

* To convert kcal to kJ, multiply it by 4·184.

† Own SES, % prof, is the % of participants in occupational social classes 1 & 2 (indicating professional occupations).

‡ Father SES, % prof, is the % of participants with father in occupational social classes 1 & 2 (indicating professional occupations).

§ Depressive symptoms calculated using the Hospital Anxiety and Depression Scale – Depression subscale score (range 1–21).

‖ Physical activity, % low, is the % of participants in the two least active groups (1, moving only in accordance with household chores, and 2, walking 1–2 times a week).

Table 3 presents the results of Cox proportional hazard models to test the associations between each of the diet scores (as continuous variables) and risk of all-cause mortality over 12 years of follow-up. In the basic-adjusted model (age, sex and energy intake), all three diet patterns were associated with risk of mortality; the MIND diet and Mediterranean-type diet were associated with a lower risk of mortality (HR 0·81; 95 % CI 0·74, 0·88, *P* < 0·001 and HR 0·74; 95 % CI 0·61, 0·89, *P* = 0·002, respectively) and the traditional diet with a higher risk of mortality (HR 1·30; 95 % CI 1·13, 1·49, *P* = 0·001).

**Table 3:** Associations between diet scores* and all-cause mortality over 12 years of follow-up, Lothian Birth Cohort 1936 study

	Model 1 basic-adjusted†			Model 2 + demographic/lifestyle-adjusted‡			Model 3 + health-adjusted§		
Dietary pattern	HR	95 % CI	*P* value	HR	95 % CI	*P* value	HR	95 % CI	*P* value
MIND	0·81	0·74, 0·88	<0·001	0·88	0·79, 0·97	0·01	0·88	0·79, 0·97	0·01
Mediterranean type	0·74	0·61, 0·89	0·002	0·88	0·72, 1·07	0·20	0·84	0·69, 1·03	0·10
Traditional	1·30	1·13, 1·49	0·001	1·19	1·01, 1·40	0·03	1·16	0·98, 1·36	0·08

MIND, Mediterranean-DASH intervention for Neurodegenerative Delay; HR, hazard ratio; SES, socioeconomic status.

* Dietary pattern scores are analysed as continuous variables.

† Model 1: adjusted for age, sex and energy intake (basic-adjusted).

‡ Model 2: additionally adjusted for age 11 IQ, education, own SES, father’s SES, smoking status, depression symptoms and physical activity (demographic and lifestyle-adjusted).

§ Model 3: additionally adjusted for BMI, hypertension, diabetes, CVD and stroke (health-adjusted).

After multivariate adjustment for demographic and lifestyle confounders (model 2), the associations with the MIND diet and traditional diet remained significant, but the Mediterranean-type diet–mortality associations were attenuated to non-significance. In the final, fully adjusted model (model 3) which additionally adjusted for health variables, only the associations with the MIND diet remained significant (HR 0·88; 95 % CI 0·79, 0·97, *P* = 0·01). Thus after adjusting for a range of potential confounders, there was a decreased risk of death in those whose diets more closely adhered to the MIND diet at baseline; risk of death was reduced by 12 % per unit increase in MIND diet score.

Additional *post hoc* analyses examined the MIND diet score as a categorical variable in the same Cox regression models in order to assess the relative risk of mortality between low, medium and high adherence groups. Using the lowest MIND tertile as the reference group, we found no significant difference between the low and medium adherence groups (HR 0·74; 95 % CI 0·52, 1·05, *P* = 0·09). However, those in the highest MIND adherence tertile had a 37 % lower risk of death (HR 0·63; 95 % CI 0·41, 0·96, *P* = 0·03) compared with those in the low tertile.

Supplemental Table 1 presents the full results of the multivariable Cox model (model 3) to show the effects of each covariate as well as the dietary pattern scores alone. Smoking (all *P* < 0·001) had the largest effect sizes across all three diet–mortality models: MIND diet (HR 2·03, 95 % CI 1·62, 2·55); Mediterranean-type diet (HR 2·08, 95 % CI 1·67, 2·59) and traditional diet (HR 2·06, 95 % CI 1·65, 2·57). Own SES, age, education, BMI, CVD and stroke also contributed to the variance in mortality across these models. Energy intake was a confounder of the traditional diet–mortality associations only.

## Discussion

In this prospective cohort study of older adults in Scotland, we found that greater adherence to the MIND diet was associated with a 12 % reduction in all-cause mortality over 12 years, per unit increase in MIND score. Individuals in the highest tertile of adherence had 37 % lower odds of premature death compared with those in the lowest group. Associations were present even after adjusting for numerous factors that correlate with a healthy lifestyle. Neither the Mediterranean-type diet nor the traditional diet patterns were significantly associated with mortality in the fully adjusted model. These findings suggest that the MIND diet, originally developed as a strategy to promote healthy cognitive ageing, may also reflect an optimal dietary pattern for prolonged survival.

To our knowledge, this is the first study to evaluate the association between adherence to the MIND diet and mortality. Conformity to the MIND diet has been demonstrated to be more effective in slowing cognitive decline and reducing incidence of Alzheimer’s disease than either the DASH or Mediterranean diets^(14,29,30)^. Our findings indicate an overall impact on health, of the MIND diet, which may extend beyond neuroprotection. Given that diet is a modifiable factor, this report is important because it has the potential to contribute to public health strategy, for the prevention of early death.

There are several biological mechanisms by which the MIND diet could impact health and survival. Although the MIND diet and the Mediterranean diet have similar dietary profiles – both share an emphasis on beneficial food components such as vegetables, fibre, wholegrains and a low red meat intake, which have independently been associated with a reduction in mortality risk^(31–35)^ – the MIND diet uniquely includes separate categories for green leafy vegetables and berries (*v*. fruit in general) which are reported to protect against neurodegeneration^(36)^. Studies on berries and mortality are rare; however, one population-based Norwegian cohort study of 10 000 men observed a significant inverse association with all-cause mortality over four decades^(37)^. In addition, other studies^(38,39)^ and a systematic review^(40)^ have detected significant risk reductions in total mortality with daily intake of green leafy vegetables. The potentially protective effects of green leafy vegetables and berries on health and survival may be explained by their potent antioxidant and anti-inflammatory properties, which is demonstrated for a number of their bioactive compounds, such as polyphenols^(41–46)^. As such, these foods may not only protect against cognitive decline but also physiological decline associated with ageing as well.

Second, unlike the Mediterranean diet score, the MIND diet score incorporates categories for less healthy food components such as ‘pastries and sweets’, ‘fast/fried foods’ and ‘butter or margarine’ due to their potentially detrimental effects on brain health^(15)^. Such foods are high in sugar, saturated fat and trans fatty acids and have an unfavourable effect on a range of health outcomes via pro-inflammatory and pro-oxidative processes^(47)^. Fast food has been shown to have a detrimental effect on future mortality risk (HR 1·16; 95 % CI 1·04, 1·29; *P* = 0·004; comparing highest *v*. lowest quartile) in a large population sample (*n* 69 582) of older adults aged 50–76 years in the VITAL study^(48)^. In the British Regional Heart Study, adopting a diet that avoids high-sugar components was associated with a reduced odds of all-cause mortality in those aged 60–79 years at baseline^(2)^ and in CHD mortality over 50 years in 12 763 men in the Seven Countries study^(49)^. Given the results of the present study, it is plausible to assume that the inclusion of healthy *and* less healthy food components in the calculation of the MIND score may be contributing to the observed associations with reduced mortality.

The Mediterranean-type dietary pattern was not significantly associated with mortality in our Scottish sample, once we accounted for a number of demographic and health factors, although prior studies have shown this diet to be strongly and inversely associated with death^(8–10,50–58)^. The majority of these investigations used the Medi score which measures adherence to a traditional Greek Mediterranean diet^(57)^ characterised by high intake of fruit and vegetables, wholegrains, fish, olive oil, a low intake of meat (white and red) and dairy products, and moderate alcohol consumption. Previous studies of older adults support a protective effect of the Mediterranean diet on all-cause mortality in Mediterranean^(9,57,58)^ and non-Mediterranean populations^(52,53,59,60)^. However, evidence from a recent meta-analysis suggests that the reduction in mortality may be weaker in countries outside of the Mediterranean basin^(7)^. Yet in the UK, where typical consumption of specific Mediterranean diet foods may be lower, high compared with low Mediterranean diet score was related to a 22 % decreased risk in overall mortality in adults aged 65 years and older (*n* 972) in the British Diet and Nutrition Survey^(59)^.

Methodologic differences in Mediterranean score derivation may be one explanation for our null finding; in the LBC1936, adherence to the Mediterranean-type pattern was assessed using an *a posteriori* score using whole-diet data. Dietary patterns derived from data reduction methods have the advantage of considering additive and interactive effects of all dietary constituents^(61)^. *A priori* defined Mediterranean diet scores, on the other hand, quantify how closely a person’s diet conforms to a set of pre-defined guidelines. The most widely used scoring system was created by Trichopoulou *et al.* ^(57)^. Both are valid approaches for determining Mediterranean diet adherence^(62)^, but one consequence of this is that comparisons between studies can be problematic. Additionally, a 2016 review paper reported that most prospective studies use sample-specific intake thresholds to measure adherence (by percentile categories)^(36)^, and therefore, similar scores can reflect different eating patterns across different cohorts. In the current study, neither wine nor fruit were prominent features of the Mediterranean-type dietary pattern (with observed factor loadings of <0·30) which contrasts with the *a priori*-derived Mediterranean score. Nonetheless, we note that the Mediterranean-type diet, as defined in our analyses using whole-diet data, was characterised by other food loadings typical of a Mediterranean diet, including a variety of vegetables, fish, legumes and olive oil. Furthermore, we observed a trend for reduced mortality with increasing Mediterranean-type diet adherence, though this association was not robust to multivariate adjustment.

In this report, we also sought to examine the relationship between a less healthy, traditional diet – characterised in our sample by a high intake of meat and processed meats, pies, potatoes, tinned vegetables and milk puddings – and mortality. Though low adherence to either of the healthy diets could be considered a low-quality diet, many different foods are consumed in a traditional diet that are not assessed in the Mediterranean or MIND diets. Therefore, it is important to examine its impact. Even within the UK, the Scottish diet is of poorer quality than in England or Wales in terms of salt, fat content, dietary cholesterol and fruit and vegetable consumption^(63)^. However, the consumption of processed foods is increasing worldwide, and in countries where the overall consumption of plant-based foods is higher, eating processed foods of low nutritional quality is associated with an increased hazard for total mortality^(64,65)^. In the SUN cohort study in Spain, high consumption of ‘ultra-processed’ foods (>4 servings daily) significantly increased mortality risk by 62 %^(65)^. In the present study, the attenuation of the association of the traditional (processed) Scottish diet and increased odds of mortality, by factors such as lower SES, less education and smoking, suggests that a low-quality/low-nutrient diet and premature death are likely connected via sociodemographic pathways across the lifecourse^(66)^. The traditional diet pattern and mortality association was also attenuated by baseline morbidity, particularly CVD and stroke.

Longevity is a multi-factorial phenomenon; a host of innate and environmental factors influences physiological ageing. In general, our study additionally highlights the strong contribution of smoking to the diet–mortality relation, an established, and dose-response, risk factor for premature death in people 60 years and older^(67)^. Despite our observation of socio-economic variation in MIND diet scores – the highest adherence group had more education, a more professional occupation and other healthy lifestyle indicators, compared with those in the lowest group – it would appear that the association between the MIND diet and mortality is less likely due to confounding, given that our risk estimates were adjusted for a range of variables, known to correlate with both diet uptake and mortality. Furthermore, risk estimates were essentially unchanged after adjustment for health status, such as obesity, CVD and stroke, potential mediators of the diet-mortality association.

### Study strengths and limitations

Some limitations of the study should be noted. One limitation involves the potential for measurement error and recall bias in FFQ. However, FFQ have been demonstrated to be the most appropriate method in assessing habitual diet in large cohorts^(68)^. It is also important to note that a high Mediterranean-type diet score does not reflect closer adherence to a true Mediterranean diet *per se*, especially in a non-Mediterranean population. We were unable to assess cause-specific mortality limiting the conclusions drawn with regard to the influence of diet on, for example, risk of cardiovascular, cancer or other deaths. Given that we present results of an observational study, we cannot infer causation or rule out residual confounding. We note that, though randomised controlled trials have the potential to demonstrate that dietary patterns can significantly lower chronic disease risk factors or outcomes, such trials are expensive and onerous to participants; therefore, most evidence will come from observational studies^(69)^. Finally, the stability of diet over time was unknown, and we cannot discount the possibility that unmeasured confounding factors over time influenced associations between the MIND diet score and the reduced odds of early death.

Generalisability of our results may be limited due to geographical location. Scotland has its own unique diet, and therefore results may differ in other populations. Scotland also has a high rate of mortality especially from CVD, though there has been a significant decline in circulatory disease–mortality in recent years (by 68 % since 1994)^(70)^. Despite this, the LBC1936 cohort is a self-selected sample from a relatively affluent area, with an interest in participating in research, and is thus more likely to be healthier than the general population.

To the best of our knowledge, this study is the first to investigate the association between the MIND diet and mortality. Other strengths of the study include its prospective nature, the use of a well-phenotyped, narrow-age sample of older adults, a relatively long follow-up period and multiple measures of diet quality. We were able to control for several confounding variables including rarely available data on childhood IQ and father’s SES; early-life cognitive ability and SES are strongly related to diet choices and health outcomes in adulthood^(71–73)^. A validated and comprehensive FFQ was used to assess dietary exposure. In addition, we obtained official linkage registry data on mortality with high reliability.

## Conclusions

In conclusion, this study shows that closer adherence to the MIND diet was associated with a lower risk of all-cause mortality over 12 years, after controlling for a range of demographic, lifestyle and health variables. This finding suggests that diet, a modifiable lifestyle factor, has the potential to mitigate risk of premature death. Crucially, we demonstrate that the MIND diet may have wider health implications beyond neuroprotection. Given the limited evidence base for this association, further prospective population-based studies and clinical trials are required to replicate this finding.

## Supplementary material

For supplementary material accompanying this paper visit https://doi.org/10.1017/S1368980020002979. click here to view supplementary material
