# Supplementary material for: Adherence to the MIND diet is associated with 12-year all-cause mortality in older adults
Source: Public Health Nutr. 2020 Sep 3;25(2):358–67. doi: 10.1017/S1368980020002979 (PMC8883600; doi:10.1017/S1368980020002979)
Supplement: Supplementary file 1 [file S1368980020002979sup001.docx]

**Supplementary table 1**

Full results of the multivariable model (model 3) to show the associations of each covariate (entered into the models simultaneously with each dietary pattern score) on 12-year mortality

|  | **MIND diet** | **Mediterranean-type diet** | **Traditional diet** |
| --- | --- | --- | --- |
| Covariates | HR (95% CI)  P-value | HR (95% CI)  P-value | HR (95% CI)  P-value |
| Dietary pattern score | 0.875 (0.788, 0.971)  **0.012** | 0.843 (0.689, 1.031)  0.096 | 1.156 (0.983, 1.360)  0.080 |
|  |  |  |  |
| Age | 0.806 (0.672, 0.966)  **0.020** | 0.816 (0.683, 0.976)  **0.026** | 0.820 (0.685, 0.981)  **0.030** |
| Sex | 1.184 (0.861, 1.630)  0.299 | 1.219 (0.892, 1.667)  0.214 | 1.246 (0.914, 1.698)  0.164 |
| Energy, kcal day | 0.972 (0.944, 1.000)  0.052 | 0.982 (0.953, 1.011)  0.222 | 0.959 (0.930, 0.990)  **0.010** |
| Age 11 IQ | 0.968 (0.864, 1.083)  0.568 | 0.962 (0.862, 1.074)  0.493 | 0.968 (0.866, 1.081)  0.562 |
| Education, years | 1.174 (0.996, 1.384)  0.056 | 1.209 (1.029, 1.421)  **0.021** | 1.222 (1.038, 1.438)  **0.016** |
| Own SES | 1.285 (1.049, 1.575)  **0.016** | 1.305 (1.068, 1.596)  **0.009** | 1.323 (1.085, 1.613)  **0.006** |
| Father SES | 1.090 (0.9228, 1.289)  0.314 | 1.117 (0.947, 1.318)  0.189 | 1.110 (0.940, 1.311)  0.220 |
| Smoking | 2.031 (1.621, 2.545)  **<0.001** | 2.077 (1.665, 2.592)  **<0.001** | 2.059 (1.647, 2.573)  **<0.001** |
| Depressive symptoms | 1.024 (0.961, 1.090)  0.467 | 1.026 (0.966, 1.091)  0.404 | 1.030 (0.969, 1.095)  0.346 |
| Physical activity | 1.007 (0.857, 1.183)  0.932 | 0.992 (0.853, 1.153)  0.912 | 0.989 (0.851, 1.149)  0.883 |
| Body mass index | 1.044 (1.007, 1.082)  **0.018** | 1.043 (1.007, 1.079)  **0.017** | 1.041 (1.006, 1.078)  **0.021** |
| Hypertension | 1.062 (0.764, 1.475)  0.720 | 1.105 (0.803, 1.521)  0.541 | 1.117 (0.813, 1.535)  0.493 |
| CVD | 1.482 (1.059, 2.073)  **0.022** | 1.505 (1.084, 2.090)  **0.015** | 1.467 (1.056, 2.037)  **0.022** |
| Diabetes | 1.594 (0.991, 2.563)  0.054 | 1.536 (0.970, 2.433)  0.067 | 1.467 (0.904, 2.278)  0.107 |
| Stroke | 2.064 (1.138, 3.743)  **0.017** | 1.977 (1.092, 3.580)  **0.024** | 1.949 (1.077, 3.528)  **0.028** |

Abbreviations: kcal, kilocalories; SES, socioeconomic status; CVD, cardiovascular disease. Depressive symptoms calculated using the Hospital Anxiety and Depression Scale – Depression subscale.

*P*-values in boldtype are significant (*P*<0.05)
